# Perceived Work Uncertainty and Creativity During the COVID-19 Pandemic: The Roles of Zhongyong and Creative Self-Efficacy

**DOI:** 10.3389/fpsyg.2020.596232

**Published:** 2020-10-29

**Authors:** Chaoying Tang, Huijuan Ma, Stefanie E. Naumann, Ziwei Xing

**Affiliations:** ^1^School of Economics and Management, University of Chinese Academy of Sciences, Beijing, China; ^2^Eberhardt School of Business, University of the Pacific, Stockton, CA, United States

**Keywords:** creative self-efficacy, Zhongyong, perceived work uncertainty, employee creativity, COVID-19

## Abstract

Research on the relationship between work stress and employee creativity has been mixed. This study on 823 female attorneys in China identifies employee creative self-efficacy and employees’ value of Zhongyong as moderators in this relationship. In this study, work stress is assessed by the perceived work uncertainty brought on by the COVID-19 pandemic. Our study found that although Zhongyong, which involves an employee’s ambidextrous thinking, can be helpful for employee creativity, low levels of Zhongyong are better for employee creativity in an uncertain context such as the COVID-19 pandemic, due to the fact that high levels of Zhongyong result in an overemphasis on compromise and giving in when times are uncertain. Instead, low levels of Zhongyong will decrease employees’ concern about others’ acceptance in an uncertain environment. In addition, creative self-efficacy motivates employees to engage in creative efforts during times of work uncertainty. In sum, this study found that employee perceived work uncertainty brought on by COVID-19 enhances employee creativity when an employee’s value of Zhongyong is low and creative self-efficacy is high.

## Introduction

Creativity in the workplace, defined as the creation of new and useful ideas or solutions (Fisher and Barrett, 2019), has been regarded as essential to organizational innovation (Akinola et al., 2019). Thus, creating conditions that allow employees to foster creativity is a key challenge for managers. But creating these conditions can be especially difficult during times of stress when employees tend to assign a greater priority to tasks that are certain and controllable, rather than creative (Luis et al., 2020). The COVID-19 pandemic offers a particularly timely context in which to examine these issues. One month after COVID-19 was declared a pandemic, a large-scale survey of U.S. employees by a leader in on-demand mental health care revealed that 69% of employees reported that this was the most stressful time of their entire professional careers (Ginger, 2020). Given the great uncertainty in the workplace caused by the current COVID-19 pandemic, identifying ways to facilitate creativity under such challenging conditions is a critical issue for organizational leaders to manage.

The present study examines how the creative efforts of attorneys in Beijing, China, in particular, were affected by the work uncertainty brought on by the COVID-19 pandemic. During the time of the investigation, the city of Beijing was experiencing an active outbreak of the virus, and citizens’ ordinary work lives abruptly changed. As a result, the attorneys in the current sample were forced to completely alter their communication approaches. For instance, during the epidemic it became challenging for lawyers to conduct on-the-spot investigations and evidence collections. In addition, they were unable to complete court applications for on-site enforcement measures. Business law attorneys lost control over properties. Attorneys in all fields needed to adapt quickly to vastly changing work arrangements, client needs, and new regulations. All of these changes required the attorneys to engage in enhanced creative efforts in order to respond effectively to these new work challenges.

Research has begun to identify cultural values as drivers of creativity (Loewenstein and Mueller, 2016). Our study focuses on the Chinese cultural value of Zhongyong, which is an indigenous concept originating from Confucian philosophy, as a predictor of employee creativity. Whereas Zhongoyong includes multiple components, our research examines the integrated thinking component of Zhongyong, which includes individuals’ tendencies to integrate different perspectives, such as ideas, opinions, and arguments, and to maintain interpersonal harmony.

Little research has examined the relationship between Zhongyong and creativity, and the results of a few studies have been mixed. Some research has found that employees with low levels of Zhongyong exhibit low levels of creativity because they are more rigid in their thinking, which goes against the flexibility needed for creativity (Shao et al., 2018). Similarly, some research has found a positive relationship between high levels of Zhongyong and creativity (e.g., Zhou et al., 2020). It may be that Zhongyong helps people advocate persuasively for new ideas, and integrate different perspectives, which is critical for creativity. However, other research has not uncovered such a relationship (Yao et al., 2010).

In addition to the lack of conclusive evidence regarding the relationship between Zhongyong and creativity, there is a gap in the literature with regard to external validity. Most research on Zhongyong has been conducted in undergraduate student samples. The present study will examine Zhongyong in a business setting. As such, our study will help to provide some support of this indigenous concept’s external validity.

Another critical gap in the existing literature involves the lack of application of paradox theory (Pinto, 2019), which is particularly suited to the examination of creativity because it examines contradictions between competing demands, such as those experienced when engaging in creative tasks. For instance, creative efforts require both divergent and convergent modes of thinking. Paradox theory has generally not been applied in creativity research to examine the role of individual differences such as creative self-efficacy (with the exception of Shao et al., 2019) and the organizational context, such as high uncertainty. Our study aims to explain the inconsistent findings in earlier research on the Zhongyong-creativity relationship by examining these variables in the same model. Our study examines the relationship between Zhongyong and creativity in an uncertain context brought on by the COVID-19 pandemic. We propose that employees with high levels of Zhongyong constantly strive to reach compromises, which might preclude their ability to generate creative ideas, in comparison with their low Zhongyong counterparts, because they tend to consider others’ interests. In an uncertain context, the risk accompanying creative behaviors and solutions would not be welcomed by most people. Our study also proposes that creative self-efficacy will play an important role in such a context, because it will motivate employees to engage in creative efforts during times of work uncertainty. The purpose of our study is to empirically test a model that proposes that employee perceived work uncertainty brought on by the COVID-19 pandemic will increase employee creativity when an employee’s value of Zhongyong is low and creative self-efficacy is high.

### Perceived Work Uncertainty and Creativity

Given that 83% of employees have reported experiencing stress at work, and have cited stress as a major source of psychological and physical problems (American Institute of Stress, 2017), it is not surprising that workplace stress has been afforded increasing attention from organizational behavior researchers. The COVID-19 pandemic has made workplace stress even more salient, as economic and social pressures mount (Ginger, 2020). In this study we examine a particularly timely component of workplace stress: perceived work uncertainty, defined as perceptions about the degree of uncertainty in relation to job characteristics and the broader work context (Leach et al., 2013).

While little research has explicitly examined the relationship between work uncertainty and creativity, research on creativity and broader measures of work stress is beginning to accumulate. Overall, the research on stress and creativity has been mixed. Studies have reported negative (e.g., Khedhaouria et al., 2017), positive (e.g., Ohly and Fritz, 2010), and curvilinear (e.g., Byron et al., 2010) relationships between stress and creativity.

The mixed findings in the existing stress-creativity literature may be explained, in part, by the conservation of resources (COR; Hobfoll et al., 2018) model of stress, which suggests that employees try to conserve their resources and obtain more resources in all situations. As a result, the threat of losing resources is the biggest contributor to stress at work. The COR model acknowledges that people vary in their ability to deal with resource loss and gain, depending on their initial levels of resources. Thus, in some studies, the initial levels of resources may have been greater, enabling employees to have enough resources to engage in creativity. However, in other contexts, employees may not have had enough initial resources to be able to expend additional resources producing creative ideas. Given the unprecedented negative effect of the COVID-19 pandemic on the resources of the workforce (Ginger, 2020), we propose the following:

Hypothesis 1: Perceived work uncertainty will be negatively associated with employee creativity.

The Moderating Effect of Zhongyong in the Relationship between Work Uncertainty and Creativity As mentioned previously, a key part of the Zhongyong orientation involves people’s tendencies to integrate ideas, perspectives, and arguments. It encourages efforts toward harmonious social interactions because it focuses on a holistic view and the balance between extremes (Yao et al., 2010). In an individual context, this manifests itself in practicing self-discipline, and searching for compromises in everyday interactions (Yao et al., 2010). The “middle-way thinking” of Zhongyong has long been viewed in China as one of the most critical meta-cognitive factors that regulate people’s emotions and beliefs (Ji et al., 2010). Indeed, this cultural value is a key part of Chinese daily life, and has been viewed as a worthy value to pursue in Chinese culture (Yang, 2010).

Multiple components of Zhongyong have been identified by researchers. For instance, Zhou et al. (2019) distinguished between four types: (1) A and B, which refers to A, but with B taken into account (e.g., the employee is ambitious, yet a team player), (2) Both A and B, which refers to A and B at the same time (e.g., the employee is proficient in both word processing and spreadsheets), (3) neither A nor B, which refers to the opposite of both A and B (e.g., the employee does not favor the union nor management), and (4) A, yet not A, which refers to having characteristics of A, but with A being prevented from being excessive (e.g., the employee is confident without being arrogant). With regard to the four types of Zhongyong, “A and B” and “Both A and B” involve integrative thinking, whereas “Neither A nor B” and “A, yet not A” capture eclectic thinking. Zhou et al. (2019) found that integrative thinking is more conducive to creative problem solving than eclectic thinking.

Research on the integrated thinking component of Zhongyong has begun to accumulate. For example, in a sample of undergraduate students, Chang and Yang (2014) investigated how differences in Zhongyong produced differences in cognitive processing styles. They found that those with a high level of Zhongyong had a larger information processing capacity compared with those with a low level of Zhongyong. The authors reasoned that the high Zhongyong individuals processed information in a more integrated and efficient manner. An implication from this research is that those with a high Zhongyong orientation deal with people and things in a global and flexible way. Other research has examined Zhongyong as a moderator of relationships between perceptual variables. Wei et al. (2020) found that a Zhongyong orientation moderated the relationship between entrepreneurial self-efficacy and job satisfaction. The scholars reasoned that Zhongyong thinking plays an important role in interpreting the cognition and behavior of Chinese entrepreneurial groups. They argued that Zhongyong is particularly helpful for entrepreneurs to manage stress and integrate resources.

Although little research has directly examined the relationship between Zhongyong and creativity, Zhang and Gu (2015) found that the moderation thinking component of Zhongyong was positively associated with employee satisfaction and creativity. Further, in a sample of college students and alumnae, Zhou et al. (2020) found that the integrated thinking component of Zhongyong was associated with a greater level of creative solutions to market investment problems. Yu and Wang (2019) suggested that, because Zhongyong thinking involves approaching a problem from more than one side and promotes a cooperative, global perspective, it is likely to be associated with innovative behavior. It has also been proposed that a Zhongyong orientation encourages individuals to exchange information and, thus, boosts innovative efforts (Wei et al., 2020).

Paradox theory (Smith and Lewis, 2011) may be used to explain why Zhongyong should be particularly useful in boosting creativity. According to the theory, paradoxical tensions are defined as contradictions between competing demands. Paradox theory is especially relevant to the study of creativity, which inherently involves competing demands. For instance, producing creative ideas involves both divergent and convergent thinking (Miron-Spektor and Erez, 2017), as well as both cognitive flexibility and cognitive persistence (Nijstad et al., 2010). However, as noted earlier, producing creative ideas can be especially challenging during times of stress when employees tend to assign a greater priority to tasks that are certain and controllable, rather than novel. Some research has begun to look at the role that cultural values such as Zhongyong play in work stress. Zhongyong has been viewed as a cognitive strategy to effectively adapt to uncertain and rapidly changing situations (Wei et al., 2020). A study by Chou et al. (2014) included a sample of nearly 400 employees in Taiwan. The authors distinguished between challenge-related stress, which involves job demands perceived by individuals as developmental opportunities, and hindrance-related stress, which involves obstacles that hinder someone’s ability to reach valued goals. They found that both types of stress resulted in emotional exhaustion, however, hindrance-related stress exhibited significantly stronger effects. The study also found that those with a low level of Zhongyong values exhibited a significant positive relationship between challenge-related stress and emotional exhaustion; in contrast, those with a high level of Zhongyong values exhibited a significant positive relationship between stress and job satisfaction (Chou et al., 2014). The authors explained their results by suggesting that employees’ Zhongyong values may lessen the negative effects of hindrance-related stress on emotional exhaustion and job satisfaction. Clearly the level of Zhongyong makes a difference in how stress affects individuals.

Given that those with high Zhongyong values tend to work toward compromises between extremes, we believe that they will be less likely to maximize their own interests and, as a result, may be quick to abandon their own opinions (Yao et al., 2010). One study involving Chinese employees spanning a diverse set of industries provided evidence that people higher on Zhongyong were less able to turn their creative ideas into innovations (Yao et al., 2010). The authors explained their findings by suggesting that those individuals who constantly strive to reach compromises are not able to advocate effectively for their unique ideas. Those with a high Zhongyong orientation prefer to empathize with others, rather than focus on their own interests (Chou et al., 2014). They value harmony, which would prevent them from proposing any interests that are incompatible with those of others because these individuals believe that opposing opinions should instead be integrated (Chou et al., 2014). Indeed, those with a strong Zhongyong orientation prefer to avoid conflicts, no matter how practical or innovative their ideas may be (Chen and Chung, 1994).

In our study, we expect that a high level of Zhongyong will prevent employees’ efforts toward creativity during a period of work uncertainty. Particularly in the current sample of attorneys experiencing significant work uncertainty due to the COVID-19 pandemic, those with low levels of Zhongyong should be less burdened by compromising too much in order to arrive at creative solutions. Various situational factors make the paradoxical tensions salient such as change or resource scarcity (Smith and Lewis, 2011), and certain individual factors are likely to play a key role in whether these tensions result in positive outcomes such as creativity or negative outcomes such as anxiety (Shao et al., 2019). To be creative, employees need not only cognitively flexibility but also persistence in maintaining their arguments, if they are to arrive at a creative solution. In contrast with the compromising style of conflict management, those with a low level of Zhongyong are likely to be unburdened by other people’s opposing attitudes toward work creativity. They are less likely to struggle with having to integrate different attitudes, which is very difficult in a period of work uncertainty, such as during the COVID-19 pandemic. As such they do not perceive the need to give up too much. A similar study suggested that a moderate Zhongyong orientation enables employees to objectively assess their uncertain work situation, and adaptively cope with the uncertainty by using introspection (Yang, 2010). Thus, we believe that high levels of the cultural value Zhongyong will prevent employees from generating creative ideas in a period of work-related uncertainty.

On the other hand, the divergent thinking brought about by the integrative thinking of Zhongyong might be limited during a period of work uncertainty. Indeed, during a crisis, the prevailing mood is negative, and it was found that negative moods may diminish divergent thinking (Baas et al., 2011). Hence, during the crisis of the COVID-19 pandemic, the positive side of Zhongyong might be limited. Thus, we propose the following hypothesis:

Hypothesis 2: Zhongyong will negatively moderate the relationship between perceived work uncertainty brought on by the COVID-19 pandemic and employee creativity, such that work uncertainty will decrease employee creativity when employees have high levels of Zhongyong.

### The Moderating Effect of Creative Self-Efficacy in the Relationship Between Work Uncertainty, Zhongyong, and Creativity

We further propose that when employees have high levels of creative self-efficacy, their low levels of Zhongyong will boost employees’ motivation to search for creative solutions during the work uncertainties of the COVID-19 pandemic, and turn this tension into motivation to engage in creativity. Creative self-efficacy, defined as one’s beliefs about one’s skills and abilities to produce creative outcomes (Farmer and Tierney, 2017), is believed to enhance intrinsic motivation toward creative efforts, and has shown a positive relationship with employee creativity (Grosser et al., 2017). Little research has examined creative self-efficacy in highly stressful contexts (Shao et al., 2019). But it has been suggested that, in situations where work-tensions run high, those employees who are able to maintain a high level of creative self-efficacy are more likely to produce more creative ideas (Shao et al., 2019). During the crisis period, the confidence derived from one’s ability to produce creativity will give employees more courage to take on the risk of generating and promoting creative ideas. In sum, we predict that whether work-related uncertainty results in higher levels of employees’ creative ideas (offsetting the negative impact of stress) depends on individuals’ perceptions of a cultural value (Zhongyong), as well as their perceptions of their abilities (creative self-efficacy). Hence, we offer the following hypothesis below:

Hypothesis 3: Employee perceived work uncertainty brought on by the COVID-19 pandemic will increase employee creativity when an employee’s value of Zhongyong is low and creative self-efficacy is high.

**FIGURE 1:**
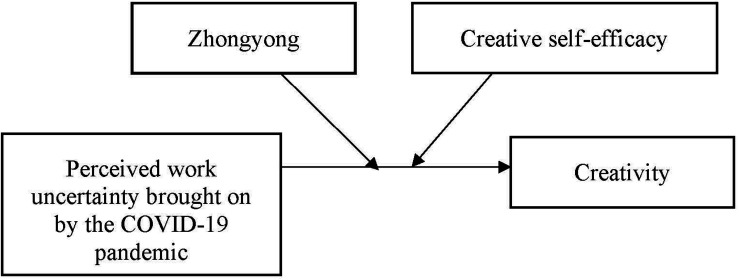
Research Model.

Our proposed model appears in Figure 1.

## Materials and Methods

### Participants and Procedure

The sample of this study includes attorneys in Beijing, China. In order to be successful, attorneys need creativity in handling litigations, such as preparing litigation plans and court debates. During the COVID-19 pandemic, attorneys were required to carry out their normal work activities, and adjust to a continually changing workload that required innovative responses. Thus, the sample was particularly relevant to the research questions in the current study.

An Association of Female Lawyers and Law Firms agreed to allow their member attorneys to participate in the current study. Between May and June 2020, the female lawyers were asked to complete an online questionnaire. Participants were assured that their responses were voluntary and anonymous. The final sample included a total of 823 complete questionnaires.

Demographic variables collected include age, tenure, education level, whether the participant was a partner in the law firm, source of income, the nature of the work, and the law firm type (Table 1). As the table shows, the average age was 39 with a range from 23 to 72 years old. 49.3% of the lawyers held a master’s degree, and 49.1% held an undergraduate degree. The average tenure of the female lawyers was 11 years.

**TABLE 1:** Sample.

	Range	N	%
Age	23–72 years	38.67	8.119
Gender	Male	0	0
	Female	823	100%
Working tenure	< 5 years	374	45.4
	5–10 years	184	22.4
	> 10 years	265	32.2
Education	Undergraduate	404	49.1
	Graduate	406	49.3
	Ph.D.	13	1.6
Partner of law firm	No	595	72.3
	Yes	228	27.7
Salary based lawyer	No	567	68.9
	Yes	256	31.1
Performance pay lawyer	No	506	61.5
	Yes	317	38.5
Second job lawyer	No	814	98.9
	Yes	9	1.1
Law firm type	Partner law firm	575	69.9
	Special partner law firm	152	18.5
	Private law firm	81	9.8
	Other provinces and cities stationed in Beijing	15	1.8

### Measures

The measures were adapted from English instruments, using a back translation procedure (Brislin, 1986) to convert to Mandarin Chinese. Survey responses were based on a Likert-type scale ranging from 1 (strongly disagree) to 5 (strongly agree). The questionnaire is available from the first author upon request.

#### Perceived Work Uncertainty Brought on by the COVID-19 Pandemic

We created a perceived work uncertainty scale loosely adapted from that of Leach et al. (2013). Whereas Leach et al. (2013) scale includes context-specific items about whether one perceives uncertainty regarding the consistency of one’s suppliers, equipment, materials, order of tasks, etc., we examined employee perceptions about uncertainty more broadly in terms of the pandemic’s effects on job stability, safety, and prospects. Three items (α = 0.78) created specifically for the current study were used to assess work stress brought on by the COVID-19 pandemic. The items included the following: “The COVID-19 pandemic makes you feel that your work is unstable”; “The COVID-19 pandemic makes you feel that your job prospects will change”; “Under the COVID-19 pandemic, you feel that your job is not safe”; “COVID-19 makes you feel unstable in your work.”

#### Creativity

Three items (α = 0.87) from Tierney et al. (1999) study were used to assess creativity: “I have tried many new methods or new solutions in my work”; “I share innovative methods or solutions with my colleagues, and I also support my colleagues’ innovative practices”; “I suggest trying new methods or new schemes to perform tasks.”

#### Zhongyong

Five items (α = 0.90) from Yao et al. (2010) were used to assess Zhongyong: “I think that being reasonable is not enough to get along with others, and I need to be sensible”, “I think that there is always a limit to everything, and it is not good to go too far or fail to reach it”; “I think that I should adjust myself for the harmony of the overall situation when handling business”; “I will consider all possible situations when I do things”; “I will find a compromise solution or a balance point among different opinions.”

#### Creative Self-Efficacy

Three items (α = 0.85) from Tierney and Farmer (2002) study were used to assess creative self-efficacy: “I feel that I am good at generating novel ideas”; “I have confidence in my creative problem solving ability”; “I have a knack for developing new methods or programs at work.”

#### Control Variables

We controlled for age and education, as these variables have been found to be associated with employee creativity (Tierney and Farmer, 2002). We also controlled for other variables thought to account for differences in creativity among attorneys: (1) Tenure, because more experienced attorneys might have accumulated more expertise, and that will influence their creativity. (2) Whether the participant was a partner in the law firm. We imagine that having a greater stake in the firm will increase their motivation to do creative work in order to increase the firm’s overall performance. (3) Source of income, which refers to whether their income is derived from a fixed amount or is based on work performance, which should influence their motivation to do creative work. (4) The nature of the work, which includes civil law, criminal law, economic law, and marriage law, etc. We believe that each would involve different facets of creativity. For instance, to be creative in the area of economic law, attorneys should excel at negotiating economic and business interests. (5) The type of law firm. Some law firms are large with hundreds of employees and a very wide variety of specialties, whereas others are small with about 10 employees specializing in one area. Thus, the organizations’ accumulated areas of expertise are different, which should influence attorneys’ creativity. (6) Part-time vs. full-time. The amount of time one spends working in law might influence their work energy and creative efforts toward the job; thus, we controlled for this demographic. In all, eight variables served as control variables.

## Results

### Correlations

Means, standard deviations, and correlations appear in Table 2. There was a significant negative relationship between creative self-efficacy and perceived work uncertainty (*r* = −0.102, *p* < 0.01), and a significant positive relationship between Zhongyong and creativity self-efficacy (*r* = 0.213, *p* < 0.01), and creativity (*r* = 0.333, *p* < 0.01). Moreover, creative self-efficacy was positively associated with creativity (*r* = 0.517, *p* < 0.01).

**TABLE 2:** Descriptive statistics and correlations among variables.

	*M*	*SD*	1	2	4	5
1. Perceived uncertainty brought by COVID-19	3.4654	0.83781	0.78			
2. Zhongyong	4.1708	0.41237	0.021	0.90		
4. Creative self-efficacy	3.8048	0.58012	−0.102**	0.213**	0.85	
5. Creativity	3.9133	0.51947	−0.025	0.333**	0.517**	0.87

**p < 0.01, two-tail tests. Alpha reliabilities are reported on the diagonal.

### Exploratory Factor Analysis

The Harman single-factor test was used to test for common method variance. The results show that the variance of the first common factor is 33.581%, which is far below the 50% standard (Yong and Pearce, 2013), indicating that there is no serious common variance problem among the measured variables. Kaiser-Meyer-Olkin (KMO) and Bartlett’s sphere tests were used to verify the validity of the questionnaire structure. The results showed that the overall KMO value of the questionnaire was 0.831, *df* = 91, *p* = 0.000, indicating that the measurement tools have good structural effects and are appropriate for factor analysis (Kaiser, 1974). Varimax rotation was used to conduct a factor analysis. Four principal components were extracted (Table 3).

**TABLE 3:** Exploratory factor analysis.

	Factor			
	Zhongyong	Creative self-efficacy	Creativity	Work stress covid19
Zhongyong 2	**0.862**	0.061	0.072	0.001
Zhongyong 4	**0.832**	0.097	0.135	0.004
Zhongyong 1	**0.825**	0.062	0.108	0.025
Zhongyong 3	**0.787**	0.106	0.030	−0.011
Zhongyong 5	**0.783**	0.089	0.143	0.024
Creative self-efficacy2	0.146	**0.842**	0.205	0.004
Creative self-efficacy 1	0.127	**0.824**	0.218	−0.046
Creative self-efficacy 3	0.066	**0.805**	0.160	−0.135
Creativity 2	0.134	0.200	**0.886**	−0.007
Creativity 3	0.215	0.157	**0.866**	−0.025
Creativity 1	0.072	0.468	**0.718**	0.037
Perceived work uncertainty brought by covid19 1	0.051	0.009	−0.081	**0.844**
Perceived work uncertainty brought by covid19 3	−0.007	−0.195	0.002	**0.825**
Perceived work uncertainty brought by covid19 2	−0.010	0.016	0.066	**0.786**
Explanation%	32.583	17.783	14.017	7.933

*Factor loadings of the four extracted principal components appear in bold.*

### Confirmatory Factor Analysis

In order to analyze the discriminant validity of the variables, we used AMOS 17.0 to conduct a confirmatory factor analysis (CFA) on the four constructs of perceived work uncertainty, creative self-efficacy, Zhongyong and creativity. According to Kline (2005), χ^2^/*df* should be <3. For the Root Mean Square Error of Approximation (RMSEA) index, it is assumed that values <0.01 indicate a perfect fit of the model to the data, and values <0.05 indicate a good fit (Hu and Bentler, 1999; Marsh et al., 2004; Hooper et al., 2008). We also examined several goodness-of-fit indices: Comparative Fit Index (CFI), Tucker Lewis Index (TLI), and Normed Fit Index (NFI). The CFI and TLI values should exceed 0.90 or even 0.95 (Hu and Bentler, 1999; Hooper et al., 2008), and the NFI should exceed 0.9 (Hair et al., 2006).

The results appear in Table 4. The structural fit statistics of the moderating model are as follows: χ^2^/*df* = 2.976, RMSEA = 0.049, RMSEA = 0.049, RMR = 0.004, CFI = 0.994, TLI = 0.976, NFI = 0.991. When we combined all four variables into one variable, the fit of the one–factor model was as follows: χ^2^/*df* = 5.340, *p* < 0.01, RMSEA = 0.073, CFI = 0.952, TLI = 0.933, NFI = 0.942. We then combined perceived work uncertainty, Zhongyong, and creative self-efficacy into one factor and kept creativity as a separate variable. We compared the fit of the two factor model with the more parsimonious three-factor, two-factor, and one-factor models to the data. The fit of the two-factor model is as follows: χ^2^/*df* = 3.117, *p* < 0.01, RMSEA = 0.051, CFI = 0.976, TLI = 0.967, NFI = 0.966. We then combined creative self-efficacy and Zhongyong into one variable, and kept perceived work uncertainty and creativity separate. The fit of this three-factor model is as follows: χ^2^/*df* = 2.868, *p* < 0.01, RMSEA = 0.048, CFI = 0.979, TLI = 0.971, NFI = 0.968. The fit of the four-factor model is as follows: χ^2^/*df* = 2.788, *p* < 0.01, RMSEA = 0.047, CFI = 0.979, TLI = 0.972, NFI = 0.967. After examining the fit of all the models, Model 4 offered a superior fit of the data.

**TABLE 4:** Confirmatory factor analysis (CFA).

	χ^2^/*df*	RMSEA	90% CI for RMSEA	RMR	CFI	TLI	NFI
One-factor Model (PWU + ZY + CSE + C)	5.340	0.073	[0.065, 0.080]	0.042	0.952	0.933	0.942
Two-factor Model (PUW + ZY + CSE, C)	3.117	0.051	[0.043, 0.059]	0.022	0.976	0.967	0.966
Three-factor Model (PWU, ZY + CSE, C)	2.868	0.048	[0.040, 0.056]	0.023	0.979	0.971	0.968
Four-factor Model (PWU, ZY, CSE, C)	2.788	0.047	[0.039, 0.054]	0.031	0.979	0.972	0.967

*PWU, Perceived work uncertainty brought by COVID-19; ZY, Zhongyong; CSE, Creative Self-Efficacy; C, Creativity.*

### Hypothesis Testing

In order to examine the hypotheses above, we used Baer (2010)’s method of testing the three-way interaction model. That is, after adding all the control variables, we then examined the prediction of perceived work uncertainty on employee creativity. Next, we put the two moderating variables into the model. After that, the three two-way interaction terms were entered into the model. Finally, the three-way interaction term was added into the model.

The regression model’s results appear in Table 5. In Model 2, perceived work uncertainty brought on by the COVID-19 pandemic was negatively associated with creativity, but it was not significant (β = −0.027, *p* > 0.10). When we added the variables in Model 3, Zhongyong and creative self-efficacy both exerted positive effects on employee creativity (β = 0.238, *p* < 0.01; β = 0.465, *p* < 0.01, respectively). When the three interaction terms were entered into Model 4, Zhongyong and perceived work uncertainty brought by the COVID-19 pandemic negatively affected employee creativity (β = −0.066, *p* < 0.05). Finally, the quadratic-three-way interaction term was entered into Model 5, and the influence was significant and positive (β = 0.072, *p* < 0.05).

**TABLE 5:** Results of hierarchical regression analysis of hypothesized effects on creativity.

		Creativity				
	Model 1	Model 2	Model 3	Model 4	Model 5	VIF
Age	0.031	0.031	−0.007	−0.008	−0.005	1.442
Working tenure	0.003	0.004	−0.045	−0.042	−0.044	1.025
Education	0.081	0.081*	0.012	0.009	0.011	1.150
Law firm type	–0.038	−0.040	−0.044	−0.042	−0.038	1.077
Partner of law firm	0.084	0.084*	0.058	0.055	0.052	1.236
Salary based attorneys	−0.076	−0.076	−0.053	−0.055	−0.055	1.863
Performance payment attorneys	−0.028	−0.028	−0.038	−0.039	−0.039	1.486
Second job attorneys	−0.034	−0.034	0.019	0.023	0.017	1.089
Perceived work uncertainty (PWU)		−0.027	0.017	0.027	0.023	1.079
Zhongyong			0.238***	0.247***	0.249***	1.126
Creative self-efficacy (CSE)			0.465***	0.468***	0.466***	1.168
PWU* Zhongyong				−0.066*	−0.077*	1.140
Zhongyong *CSE				−0.020	−0.018	1.158
PWU*CSE				0.024	−0.015	1.559
PWU* Zhongyong*CSE					0.072*	1.623
Adj. *R*^2^	0.012	0.011	0.321	0.323	0.325	
△*R*^2^	0.021	0.001	0.308	0.004	0.003	
*F*	2.233*	0.611	186.502***	1.723	3.922*	
*df*1, *df*2	8, 814	1, 813	2, 811	3, 808	1, 807	

N = 823, *p < 0.10, ***p < 0.01, two-tail tests. Values presented are standardized regression coefficients.

**FIGURE 2:**
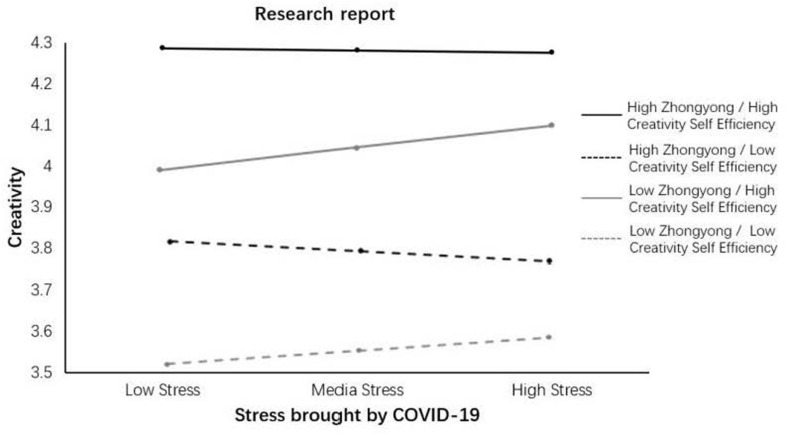
Interactions.

We divided the sample into four groups. The criteria we used was that, if the data were greater than one standard deviation above the mean, they were assigned to the high-level group. If the data were less than one standard deviation below the mean, they were assigned to the low-level group. We found that when Zhongyong is low and creative self-efficacy is high, perceived work uncertainty positively influenced creativity (β = 0.066, *p* < 0.05). When Zhongyong is low and creativity self-efficacy is at a middle level, perceived work uncertainty also positively influenced creativity (β = 0.055, *p* < 0.05). In all the other cases, perceived work uncertainty was not associated with employee creativity. The moderating effect is presented in Figure 2. Thus, Hypothesis 1 was not supported, but Hypotheses 2, and 3 were supported.

## Discussion

The COVID-19 pandemic has created challenges for employees across the world. But the results of this study show that crises such as the current one can facilitate people’s efforts toward creativity and innovation under certain conditions. Specifically, we found that employee perceived work uncertainty brought on by the pandemic enhanced employee creativity when an employee’s value of Zhongyong is low and creative self-efficacy is high.

Our study contributes to the growing body of research on Zhongyong, providing further evidence of how culture shapes behavior. We respond to Chou et al. (2014) call for more research on how employees’ Zhongyong beliefs link to creativity and stress. Most research on Zhongyong has been conducted in undergraduate student samples. The current study offers evidence that Zhongyong affects key variables in the business world as well. As such, our findings help to provide some support of this indigenous concept’s external validity.

Our first hypothesis, which proposed that perceived work uncertainty would be negatively associated with creativity, was not supported. Whereas this finding was unexpected, it is consistent with a previous study by Ohly and Fritz (2010) on employees at an auto manufacturer, which did not detect a negative relationship between these variables. The authors postulated that this finding might be sample-specific, and called for future research to identify moderators in the relationship between stress and creativity.

We also believe that the lack of a negative relationship between work uncertainty and creativity may be explained by the fact that moderators (i.e., Zhongyong and creative self-efficacy) were intervening in this relationship. Specifically, we found that employee perceived work uncertainty brought on by the COVID-19 pandemic increased employee creativity when an employee’s value of Zhongyong was low and creative self-efficacy was high, supporting our last two hypotheses. As such, our study’s findings contribute to the application of paradox theory in the literature. The current body of research on the theory ignores the role of individual differences such as creative self-efficacy (with the exception of Shao et al., 2019) and the organizational context, such as one of high uncertainty (Schad et al., 2016). Thus, we believe that one explanation for the inconsistent findings in earlier research on the relationship between Zhongyong and creativity may be that the previous works did not consider work contexts with a high level of perceived work uncertainty in their studies. The role of creative self-efficacy in the current study fits with previous arguments that feelings of self-competence lead individuals to have a desire to exert effort toward creative work (Deci and Ryan, 1985). Although little research has examined creative self-efficacy in uncertain work contexts, Shao et al. (2019) suggested that, in stressful work situations, those employees who are able to maintain a high level of creative self-efficacy are more likely to produce more creative ideas.

Our study also has implications for practice. Given the benefits of low levels of Zhongyong to employee creativity, managers should consider designing training programs to help employees learn not to over-compromise on their novel and unique ideas during a period of work uncertainty. Boosting employees’ motivation to offer new ideas also requires an organizational climate that is perceived as psychologically safe. This is particularly important when negative moods abound, such as during the COVID-19 pandemic.

In addition, given the benefits of creative self-efficacy, managers should provide their subordinates with some key examples in which employees were previously able to generate creative ideas under uncertain conditions. It has been suggested that persuasion from one’s manager helps to boost employees’ levels of creative self-efficacy (Tierney and Farmer, 2002). Giving employees positive feedback about their creative behaviors and rewarding such efforts will also help improve employees’ self-confidence in their creative abilities. Offering creativity training sessions is another useful approach to improving employees’ creative self-efficacy.

Our study is not without limitations. It involved a sample of attorneys in China. Thus, the study’s findings may not be generalizable to cultures that do not include the value of Zhongyong. For instance, Song et al. (2016) have suggested that, in comparison with U.S. citizens, Chinese people apply stronger emotional constraints on themselves and are less prone to display extremes in their perceptions. In addition, because Zhongyong considers revealing extreme emotions harmful for relationships, the researchers argued that the psychological cost for Chinese people to risk congenial relationships by expressing excessive emotions is greater than for U.S. citizens. Thus, the concept of Zhongyong may be less relevant in non-Chinese samples. Second, due to the nature of our sample being restricted to female attorneys, it did not allow for an examination of the hypotheses in a context with gender diversity. Third, our study involved self-reported creativity measures. It was not possible to include supervisor-rated or objective measures of creativity during the COVID-19 pandemic.

Future research should examine the role that cultural values such as Zhongyong in field studies on creativity around the world. In addition, we agree with Yao et al. (2010) that future Zhongyong research should use non-self-reported creativity measures to offer a more complete understanding of employee creativity.

## Conclusion

In conclusion, given the great uncertainty in the workplace caused by the current COVID-19 pandemic, identifying ways to facilitate creativity under such challenging conditions is a key problem for today’s managers. To help employees generate creative ideas under stressful work conditions, managers should train employees in low levels of Zhongyong so that they may learn to avoid compromising too much on their novel ideas. In addition, managers should try a variety of different approaches to enhance their employees’ self-efficacy.

## Data Availability Statement

The minimal data set underlying the findings described are available to any qualified researcher. Requests should be directed to tcy@ucas.ac.cn.

## Ethics Statement

The studies involving human participants were reviewed and approved by the University of Chinese Academy of Sciences. The patients/participants provided their written informed consent to participate in this study.

## Author Contributions

CT designed the research, revised the writing, analyzed the data. HM designed the research and collected data. SN wrote the abstract, introduction, literature review, discussion, references sections, and edited the other sections. ZX analyzed the data. All authors contributed to the article and approved the submitted version.

## Conflict of Interest

The authors declare that the research was conducted in the absence of any commercial or financial relationships that could be construed as a potential conflict of interest.
